# Addendum: A joint view on genetic variants for adiposity differentiates subtypes with distinct metabolic implications

**DOI:** 10.1038/s41467-018-05088-6

**Published:** 2018-07-20

**Authors:** Thomas W Winkler, Felix Günther, Simon Höllerer, Martina Zimmermann, Ruth JF Loos, Zoltán Kutalik, Iris M Heid

**Affiliations:** 1 Department of Genetic Epidemiology, University of Regensburg, D-93051 Regensburg, Germany; 2 Statistical Consulting Unit StaBLab, Department of Statistics, Ludwig-Maximilians-Universität Munich, D-80539 Munich, Germany; 3 The Charles Bronfman Institute for Personalized Medicine, Icahn School of Medicine at Mount Sinai, 10029 New York, NY USA; 4 The Genetics of Obesity and Related Metabolic Traits Program, Icahn School of Medicine at Mount Sinai, 10029 New York, NY USA; 5 The Mindich Child health and Development Institute, Icahn School of Medicine at Mount Sinai, 10029 New York, NY USA; 6 Institute of Social and Preventive Medicine (IUMSP), Centre Hospitalier Universitaire Vaudois (CHUV), 1010 Lausanne, Switzerland; 7 Swiss Institute of Bioinformatics, 1015 Lausanne, Switzerland

**Keywords:** Genome-wide association studies, Quantitative trait, Metabolic diseases

Addendum to: *Nature Communications*; 10.1038/s41467-018-04124-9; published online 16 May 2018

We would like to clarify that the odds ratios reported in Results and Discussion of this article refer to disease risk increase per unit increase in BMI, where (i) BMI is standardized to a standard normal distribution (1 unit equivalent to one standard deviation of BMI, ~4.5 kg/m^2^), and (ii) this increase in BMI refers to the specific adiposity subtype (given by the respective genetic variants’ class). This should have been specified where the odds ratios were presented in the Results and Discussion sections of this article.

