# Supplementary material for: A joint view on genetic variants for adiposity differentiates subtypes with distinct metabolic implications
Source: Nat Commun. 2018 May 16;9:1946. doi: 10.1038/s41467-018-04124-9 (PMC5956079; doi:10.1038/s41467-018-04124-9)
Supplement: Supplementary file 1 — Supplementary Information [file 41467_2018_4124_MOESM1_ESM.docx]

**SUPPLEMENTARY INFORMATION**

**A joint view on genetic variants for adiposity differentiates subtypes with distinct metabolic implications**

Winkler et al.

# SUPPLEMENTARY NOTES

## **Supplementary Note 1.** Computing variance estimates of WHR effects from meta-analyzed BMI and WHRadjBMI effects and their variance estimates.

Based on the effect for BMI and WHRadjBMI and their variance estimates, the WHR effect and its variance can be computed via $b_{WHR}=b_{WHRadjBMI}+r*b_{BMI}$ and $\hat{Var}\left( b_{WHR} \right)= \hat{Var}\left( b_{WHRadjBMI} \right)+r^{2}\hat{Var}\left( b_{BMI} \right)+ 2r\hat{Cov}(b_{WHRadjBMI}, b_{BMI})$. Here, r denotes the phenotypic correlation between WHR and BMI in the study – or the average of all included studies in a meta-analysis setting. Under the assumption of a covariance between BMI and WHRadjBMI effect close to zero (Spearman correlation coefficient between b_WHRadjBMI_ and b_BMI_ in GIANT data is <0.01), we can derive $\hat{Var}\left( b_{WHR} \right)$ as $\hat{Var}\left( b_{WHR} \right)= \hat{Var}\left( b_{WHRadjBMI} \right)+r^{2}\hat{Var}\left( b_{BMI} \right)$.

Since this computation of $\hat{Var}\left( b_{WHR} \right)$ involves the sampling error of both, the WHRadjBMI effect and the BMI effect, this computed variance estimate will be expected to be larger than the observed variance estimate. We exemplify how well this computation of $Var\left( b_{WHR} \right)$ compared to the directly estimated $\hat{Var}\left( b_{WHR} \right)$ in practice. For the 38 genome-wide significant WHR variants from GIANT, the WHR standard errors were re-calculated from BMI and WHRadjBMI GIANT meta-analysis estimates. These are on average 10.3% larger than the standard errors from the original WHR meta-analysis (**Supplementary Fig. 2**).

## **Supplementary Note 2. Visualization of WHR and BMI effect based on the co-association of waist and hip circumference or weight and height**

It is important to visualize a genetic effect on a composite phenotype like WHR or BMI by the co-association of the constituents, waist and hip circumference or weight and height, respectively. A variant shows an effect on WHR, if it affects WC and HIP disproportionally. This can derive from an effect on waist without an effect on hip, an effect on hip without an effect on waist, an effect on both into the same direction, or an effect on both into the opposite direction, but to an extent that is not expected (“disproportionally”) (**Supplementary Fig. 4**). The expected direction and extent is given by mean(WHR) in a study or the average across all involved studies. An effect that has no effect on WHR either has no effect on waist and hip or affects waist and hip proportionally as expected given the mean(WHR). When looking at the co-association of waist and hip circumference (**Fig. 4a**), the line through the origin defined by b_WC_=mean(WHR)*b_HIP_ represents the null line for WHR. When utilizing the estimate of the mean(WHR) from the CoLaus study, mean(WHR)=0.88, considering this an average estimates across all studies, the null line is defined as b_WC_=0.88*b_HIP_. For example, the fact that *BMIonly+* variants scatter around the null line illustrates the proportionality between waist and hip effects and visualizes the lack of WHR effect for variants in this class. In contrast, *WHRonly-* and *BMI+WHR-* variants are orthogonally distant from this null line. This can be compared to waist adjusted for hip (WCadjHIP), where the WCadjHIP effect, b_WCadjHIP_, is given by b_WCadjHIP_=b_WC_-r_WC,HIP_*b_HIP_, with r being the phenotypic correlation between WC and HIP. In CoLaus, we have r_WC,HIP_=0.80, and thus a similar null line for the effect on WCadjHIP compared to the effect on WHR.

Similarly, for a variant that affects BMI = WT/HT^2^, the co-association of weight (WT) and height (HT) can be visualized by a scatter plot of b_WT_ versus b_HT_ (**Fig. 4B**). The effect on BMI, b_BMI_, can be viewed as the distance of each SNP from the line through the origin defined by b_WT_=[2*mean(HT)*mean(BMI)*SD(HT)/SD(WT)]*b_HT_, with mean and SD indicating the phenotypic sample mean and sample standard deviation of an underlying study or the average across all included studies. This line represents the null line for BMI, b_BMI_=0, approximately fulfilled for b_WT_=0.54*b_HT_, using 1.69 m, 25.85 kg/m², 0.0932 m, and 15.11 kg for mean(HT), mean(BMI), SD(HT), and SD(WT) estimated from CoLaus. This null line coincides with b_WT_= 0.53 *b_HT_, which reflects a null effect of weight adjusted for height (WTadjHT) with r_WT,HT_=0.53 being the Pearson correlation coefficient between weight and height in our example study, CoLaus. This derives from the relationship b_WTadjHT_=b_WT_-r_WT,HT_*b_HT_. A WTadjHT scan will thus yield similar results as a scan for weight divided by height (squared or not), which is basically a BMI-scan.

With the same line of argument as outlined above for WHRadjBMI (**Supplementary Note 1**), a WTadjHT scan or a WT/HT² scan (i.e. BMI scan) will depict not only weight loci with an unusual height effect (considering the weight loci with expected height effect to be primary height effects and thus uninteresting for BMI genetics), but also height loci with an unusual effect on weight (e.g. height effects without directionally consistent effect on weight). A genomic scan on BMI can thus be considered a scan to identify genetic variants with effects for weight that are unexpected given the variants’ effects on height. Composite phenotypes (e.g. quotients of two measures), just like adjusted-model phenotypes, have to be interpreted with great care.

## Supplementary Note 3: Scan-specific DEPICT analyses

Scan-specific DEPICT analyses are shown in **Supplementary Fig. 5** with details in **Supplementary Data 8**. For BMI-scan derived variants, we were able to replicate the results from Locke and colleagues[^1^](#_ENREF_1): Of the 19 significantly enriched tissues, cell types and physiological systems (FDR < 0.05), 18 were part of the central nervous system. For the WHRadjBMI-scan derived variants, we replicated results from Shungin and colleagues[^2^](#_ENREF_2): Of the eight significantly enriched tissues, cell types and physiological systems (FDR < 5%), six were part of connective tissues. For the WHR-scan derived variants, we did not observe any significant enrichments (FDR ≥ 5%) suggesting that WHR is a trait that is less distinct than WHRadjBMI or BMI with regard to the underlying biological pathways.

The slight differences between our and previous (Locke, Shungin) results were due to our slightly different locus definition and due to harmonizing P-Value thresholds between BMI and WHRadjBMI (using P < 10^-5^ for all, whereas P < 5 x 10^-4^ had been used by Locke et al., and P < 10^-5^ by Shungin et al.).

## Supplementary Note 4: Estimation of effects of G on YadjZ

We were interested whether it is possible to have a genetic variant with a significant WHRadjBMI effect, where this variant has no true effect on WHR nor on BMI. Let the random variable *Y* represent an outcome trait of interest and *Z* another trait for which we wish to adjust *Y*. We further assume that a genotype *G* has a linear effect on *Y* and *Z,* but allow this effect to be zero. Assuming that *Y*, *Z* and *G* have zero mean, this corresponds to $E\left( Y | G \right)= \alpha G$, $E\left( Z | G \right)= \beta G$ and $E\left( Y | Z \right)= \gamma Z$. The adjusted trait *YadjZ* can formally be written as $YadjZ=Y-E(Y|Z)$. This implies that

$$E\left( YadjZ | G \right)= E\left( \left( Y-E\left( Y | Z \right) \right) | G \right)= E\left( Y | G \right)-E\left( E\left( Y | Z \right) | G \right)= E\left( Y | G \right)- E\left( \gamma Z | G \right)= \alpha G-\gamma E\left( Z | G \right)=\alpha G-\gamma\beta G= (\alpha-\gamma\beta)G$$

Therefore, if our data show evidence for a non-zero linear effect of *G* on *YadjZ*, we expect that $\alpha-\gamma\beta\neq0$. This implies that not both $\alpha=0$ and $\beta=0$, hence *G* has a non-zero linear effect on at least one of the two traits *Y* or *Z*. On the example of a variant with a significant WHRadjBMI effect in the data, where we deduce that there is evidence for a true non-null effect, we can expect that this variant will not have a true zero effect on both WHR and BMI.

The relationship between adjusted trait effects *YadjZ* and direct *Y* and *Z* effects as shown by Aschard and colleagues was derived under the assumption that the adjustment was done directly in the association model.

In GIANT studies, the linear regression model

$$YadjZ=\beta_{0}+\beta_{YadjZ}G+\varepsilon$$

is applied to estimate the genetic effect $\beta_{YadjZ}$ for a genetic variant G (omitting the index by study and that we combine study-specific effect estimates across studies using a fixed effect model), where $YadjZ$ are the residuals of a simple linear regression model

$$Y=\gamma_{0}+\gamma_{1}Z+\varepsilon.$$

As in any simple linear regression, the slope parameter $\gamma_{1}$is estimated by

$$g_{1}=\frac{\hat{Cov}(Z,Y)}{\hat{Var}(Z)}.$$

$YadjZ$ can therefore be expressed as

$$YadjZ=Y-g_{0}-\frac{\hat{Cov}(Z,Y)}{\hat{Var}(Z)}Z$$

and the estimated effect of $G$ on $YadjZ$ is

$$b_{YadjZ}=\frac{\hat{Cov}(G,YadjZ)}{\hat{Var}(G)}$$

$$=\frac{1}{\hat{Var}(G)}*\hat{Cov}(G,Y-\frac{\hat{Cov}(Z,Y)}{\hat{Var}(Z)}Z)$$

$$=\frac{1}{\hat{Var}(G)}*[\hat{Cov}(G,Y)-\frac{\hat{Cov}(Z,Y)*\hat{Cov}(G,Z)}{\hat{Var}(Z)}].$$

If $Y$ and $Z$ are standardized to have $\hat{Var}(Z)=\hat{Var}(Y)=1$, this reduces to

$$b_{YadjZ}=\frac{\hat{Cov}\left( G,Y \right)}{\hat{Var}\left( G \right)}-\hat{Cor}\left( Z,Y \right)\frac{\hat{Cov}\left( G,Z \right)}{\hat{Var}\left( G \right)}=b_{Y}-r\times b_{Z},$$

where $b_{Y}$ and $b_{Z}$ are the estimated slope effects of simple linear regression models of $Y$ on $G$ and $Z$ on $G$, respectively, and $r$ is the estimate of the correlation between the two traits $Z$ and $Y$. In GIANT, WHR corresponds to Y and BMI to Z.

##

# SUPPLEMENTARY FIGURES

**
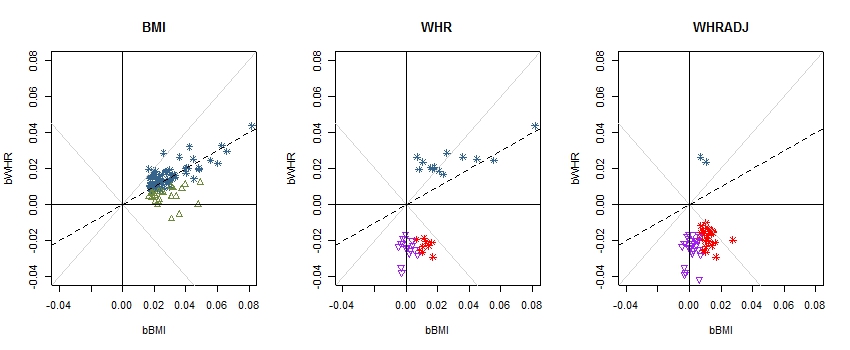
**

## **Supplementary Figure 1. Co-association of the 159 variants with WHR and BMI separated by the scan which identified the variant.** Shown are the 159 variants on the b_WHR_-b_BMI_ plane separately for the variants identified A) by the BMI-scan, B) by the WHR-scan, C) by the WHRadjBMI-scan. Color codes the four different classes (blue: BMI+WHR+, green: BMIonly+, red: BMI+WHR-, purple: WHRonly-).


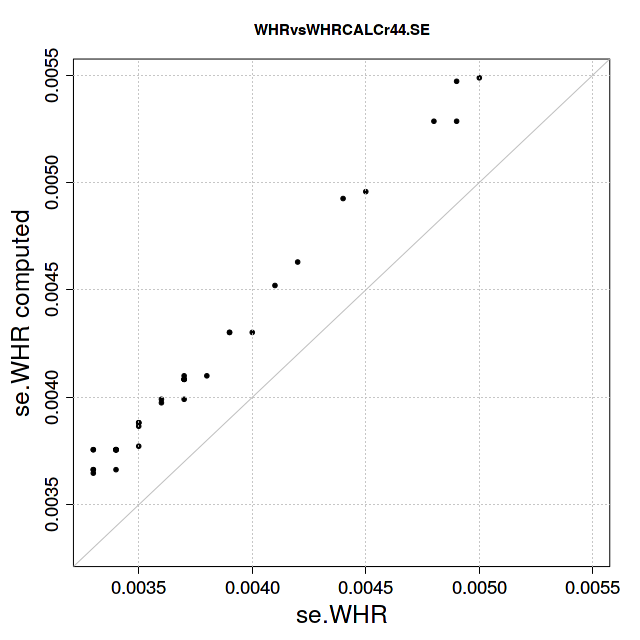


## **Supplementary Figure 2. Comparison of estimated and computed WHR standard errors for the 38 genome-wide significant WHR-derived lead variants.** Using GIANT meta-analysis summary statistics, we compare standard errors of meta-analysed overall WHR effects (resulting from meta-analysis of multiple studies) with standard errors of computed WHR effects that were calculated from meta-analysed overall BMI and WHRadjBMI association statistics using the overall correlation between WHR and BMI (r=0.44, in UKBB).


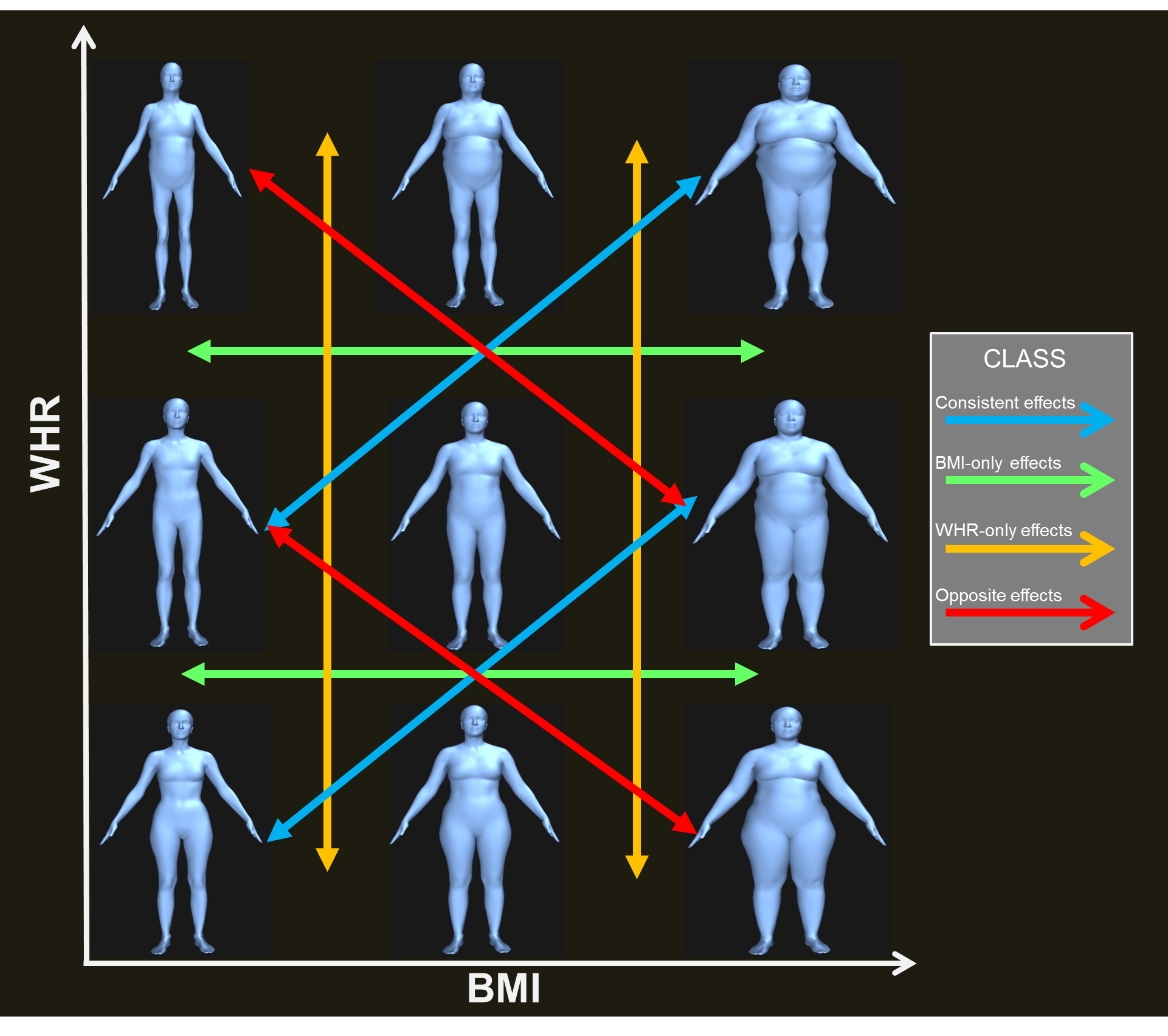


## Supplementary Figure 3. Visualization of genetic effects for their co-association with BMI and WHR. The figure shows phenotypes of persons based on their BMI and WHR (<http://bodyvisualizer.com/>) and highlights examples of genetic effects via arrows: (i) An effect of increasing BMI will expectedly (meaning “as observed on average”) come with increasing WHR (*BMI+WHR+*, blue arrow, increasing fat depots more on waist than on hip, e.g. moving from the middle person to the person on the top right). (ii) An effect of increasing BMI without any effect on WHR (*BMIonly+*, green) is an effect where the additional fat distributes in a comparable way on waist and hip. (iii) An effect of decreasing WHR without any effect on BMI (*WHRonly-*) is an effect of decreasing waist and increasing hip (orange arrow). *BMIonly+* and *WHRonly-* effects are less expected given the phenotype correlation; if all effects would be that way, the phenotypic correlation would be zero. (iv) An effect that decreases WHR, but increases BMI (*BMI+WHR-*, red arrow) is also unexpected; if all effects would be that way, the phenotypic correlation would be negative. Such an effect can be found in the figure when looking at the person in the middle and comparing her/him to the most right person on the bottom: This person exhibits increased hip (i.e. decreased WHR). This supports the importance also of hip circumference, which is missed when focussing on “central adiposity”.


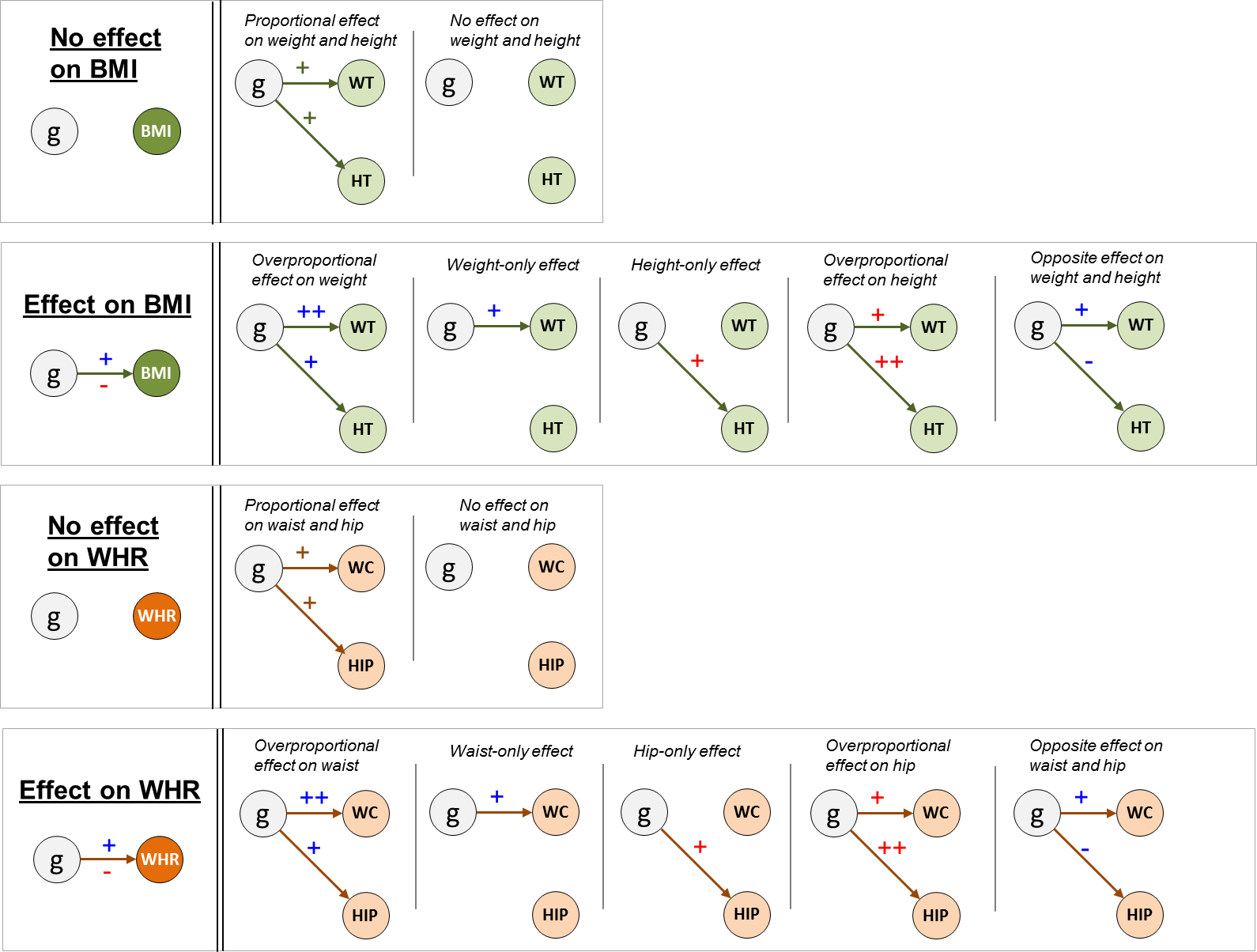


## Supplementary Figure 4. Implications of BMI and WHR effects on anthropometric measures. The figure visualizes various combinations of genetic effects on weight (WT) and height (HT) that result in zero, positive or negative genetic effects on BMI; also shown are the combinations of genetic effects on waist circumference (WC) and hip circumference (HIP) that yield zero, positive or negative genetic effects on WHR. Blue/red signs indicate a positive/negative effect on BMI or WHR. It is important to note that null effects on BMI or WHR can derive from proportional effects on both constituents (WT and HT or WC and HIP).


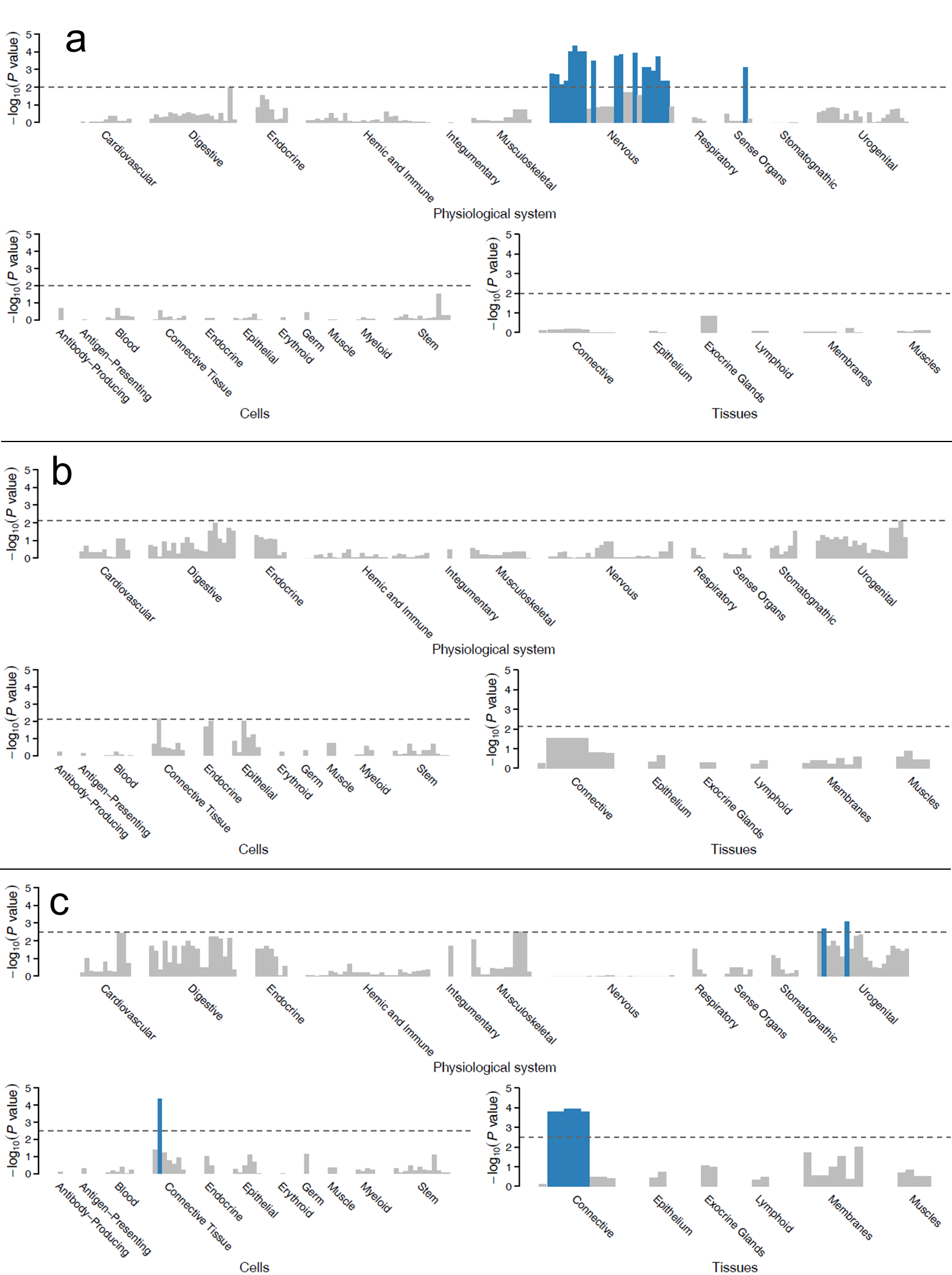


## Supplementary Figure 5. Scan-specific enrichment of gene expression. Shown are the results of the scan-specific DEPICT analysis based on variants identified from a: the BMI-scan, b: the WHR-scan, or c: the WHRadjBMI-scans (P<10^-5^ in the respective GIANT meta-analysis excluding metabochip). DEPICT evaluates expression pattern in 37,427 human microarray samples; shown are significantly enriched signals grouped by type and ordered alphabetically by MeSH term within a specific system, cell type or tissue (blue bars denote significance, FDR<5%, details in Supplementary Data 8).

**
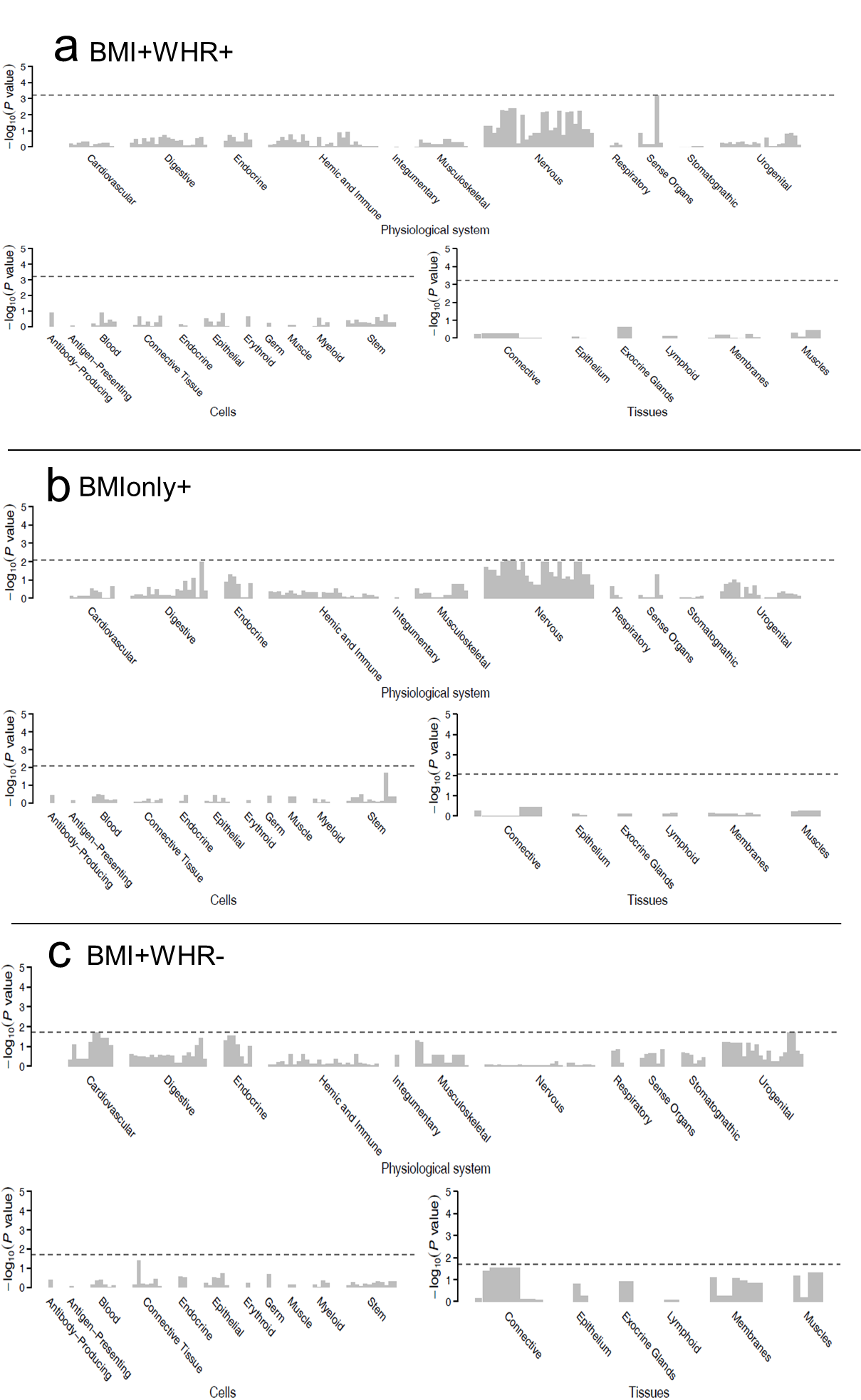
**

## Supplementary Figure 6. Class-specific tissue-specific enrichment of gene expression using DEPICT. Shown are the results of the class-specific DEPICT analyses based on variants that were selected from GWAS-only meta-analyses of GIANT (P<10^-5^) and classified as a: *BMI+WHR+*, b: *BMIonly+*, c: *BMI+WHR-*. For. *WHRonly-*: please see Fig. 6. Results are grouped by type and ordered alphabetically by MeSH term within a specific system, cell type or tissue (no finding was significant to FDR<5%, details in Supplementary Data 9).

**
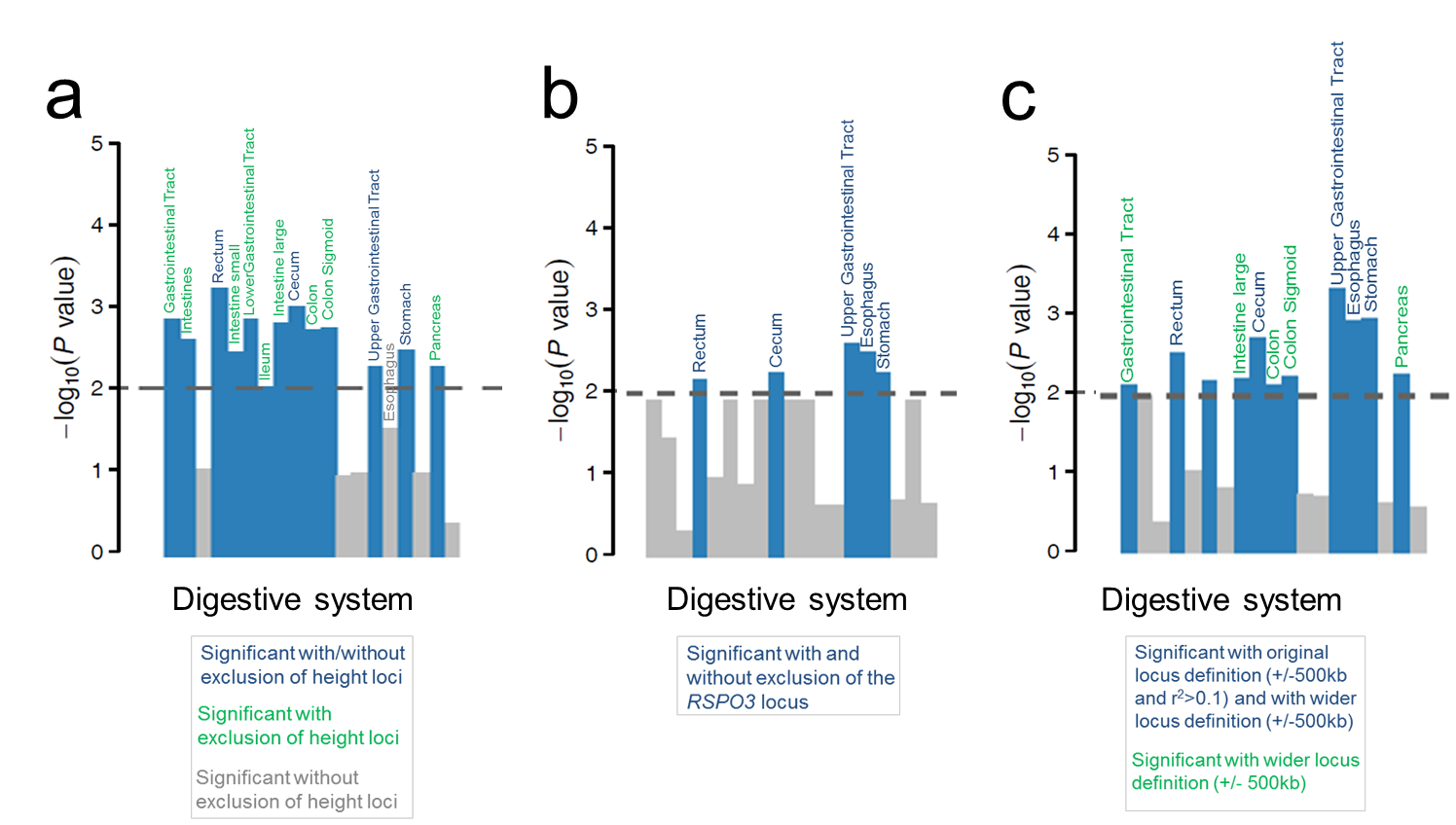
**

## Supplementary Figure 7. DEPICT sensitivity analyses for *WHRonly-* class loci. Shown are the DEPICT results of sensitivity analyses for the variants in the *WHRonly-* class a: excluding 697 genetic regions known to be associated with human height (Wood et al) (removing all WHR-only variants within <250kB around the lead variants) yielding nine additional significant enrichments, b: excluding any variants harbouring the *RSPO3* locus - yielding similar results, and c: using a wider locus definition criterion that is based on distance-only (+/-500kb, no additional r^2^ criterion) - yielding five additional significant enrichments. Blue bars indicate significant enrichments (FDR<5%). Results are ordered alphabetically by MeSH term. Detailed results are shown in Supplementary Data 10-12.


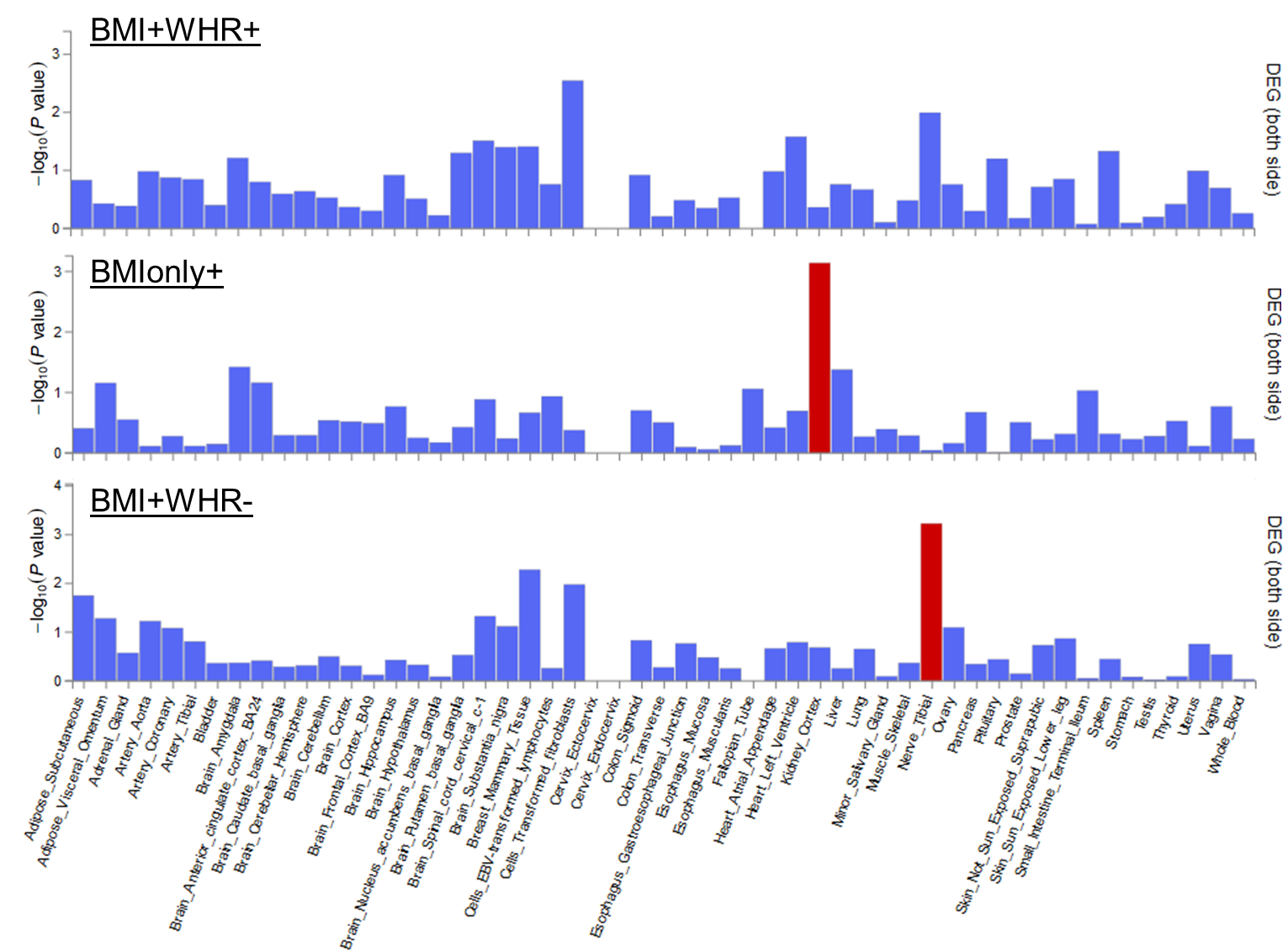


## Supplementary Figure 8. Class-specific tissue-specific enrichment of gene expression using FUMA. Shown are the results of the class-specific FUMA analyses based on variants that were selected from GWAS-only meta-analyses of GIANT (P<10^-5^) and classified as *BMI+WHR+*, *BMIonly+* or *BMI+WHR-* (details in Supplementary Data 13). For*. WHRonly-*, please see Fig. 6. Significant enrichments are highlighted in red (adjusted P<0.05, Bonferroni-corrected). The -log10(P values) in the graph refer to the probability of the hypergeomteric test.

# SUPPLEMENTARY TABLES

## Supplementary Table 1. Results of class-specific meta-regression based genetic risk score analyses. The table shows results from class-specific meta-regression analyses that were conducted to infer the joint genetic risk (weighted by the BMI-effect) of variants of each class on various traits including anthropometric traits, impedance fat measures, ectopic fat traits and cardio-metabolic traits and disease. The table shows the number of variants used in the meta-regression (mSNPs), the estimated joint effect (a) with standard error (se) and respective P-Value (P_GRS_). Bold P-Values indicate significant joint effects (P_GRS_<0.05/60, Bonferroni-corrected for 60 meta-regressions).

|  |  | ***BMI+WHR+*** | | | | ***BMI-only*** | | | | ***BMI+WHR-*** | | | |
| --- | --- | --- | --- | --- | --- | --- | --- | --- | --- | --- | --- | --- | --- |
| Trait | N | mSNPs | a | se | P_GRS_ | mSNPs | a | se | P_GRS_ | mSNPs | a | se | P_GRS_ |
| *Anthropometric traits:* | |  |  |  |  |  |  |  |  |  |  |  |  |
| WHR | 212,216 | 82 | 0.546 | 0.015 | **5.7E-283** | 25 | 0.171 | 0.035 | **1.28E-06** | 24 | -1.480 | 0.064 | **5.3E-118** |
| WC | 232,083 | 82 | 0.908 | 0.016 | **0** | 25 | 0.716 | 0.036 | **1.89E-88** | 24 | 0.048 | 0.066 | 0.464 |
| HIP | 213,028 | 82 | 0.853 | 0.016 | **0** | 25 | 0.885 | 0.038 | **9.58E-123** | 24 | 1.340 | 0.068 | **1.59E-85** |
| HT | 253,239 | 82 | 0.042 | 0.013 | 0.00161 | 25 | -0.034 | 0.031 | 0.272 | 24 | -0.279 | 0.057 | **1.01E-06** |
| WT | 125,943 | 82 | 0.964 | 0.020 | **0** | 25 | 0.936 | 0.048 | **5.09E-86** | 24 | 0.802 | 0.087 | **1.89E-20** |
| *Impedance measures:* | |  |  |  |  |  |  |  |  |  |  |  |  |
| Body fat | 114,178 | 80 | 7.170 | 0.171 | **0** | 25 | 6.080 | 0.381 | **3.47E-57** | 24 | 6.230 | 0.716 | **3.11E-18** |
| Trunk fat | 114,305 | 80 | 3.860 | 0.096 | **0** | 25 | 3.340 | 0.214 | **6.01E-55** | 24 | 3.490 | 0.402 | **3.57E-18** |
| Leg fat | 114,367 | 80 | 2.270 | 0.056 | **0** | 25 | 1.920 | 0.124 | **4.12E-54** | 24 | 1.810 | 0.232 | **6.75E-15** |
| *Ectopic fat traits:* | |  |  |  |  |  |  |  |  |  |  |  |  |
| VAT | 18,312 | 82 | 0.537 | 0.048 | **3.01E-29** | 24 | 0.315 | 0.110 | 0.00403 | 24 | -0.579 | 0.200 | 0.00377 |
| SAT | 18,206 | 82 | 0.739 | 0.048 | **1.46E-53** | 25 | 0.629 | 0.108 | **5.85E-09** | 24 | 1.090 | 0.200 | **5.14E-08** |
| VAT/SAT | 18,205 | 82 | -0.142 | 0.048 | 0.00312 | 25 | -0.224 | 0.108 | 0.0378 | 24 | -1.900 | 0.200 | **2.04E-21** |
| PAT | 11,616 | 82 | 0.328 | 0.062 | **9.55E-08** | 25 | 0.153 | 0.138 | 0.267 | 24 | -1.300 | 0.254 | **2.96E-07** |
| *Cardio-metabolic traits and diseases:* | | | |  |  |  |  |  |  |  |  |  |  |
| CAD | ~185,000 | 81 | 0.424 | 0.043 | **1.72E-23** | 25 | 0.194 | 0.097 | 0.0448 | 23 | -0.849 | 0.180 | **2.50E-06** |
| MI | ~170,000 | 81 | 0.377 | 0.047 | **1.34E-15** | 25 | 0.223 | 0.108 | 0.0382 | 23 | -0.772 | 0.201 | **0.00012** |
| HDL-C | 187,135 | 81 | -0.259 | 0.016 | **9.10E-61** | 25 | -0.218 | 0.036 | **1.99E-09** | 24 | 0.913 | 0.066 | **1.18E-43** |
| LDL-C | 173,058 | 81 | -0.038 | 0.017 | 0.0261 | 25 | -0.052 | 0.039 | 0.184 | 24 | -0.329 | 0.070 | **2.99E-06** |
| TG | 177,829 | 81 | 0.222 | 0.015 | **2.46E-47** | 25 | 0.117 | 0.035 | 0.000914 | 24 | -0.961 | 0.064 | **4.78E-51** |
| T2D | 69,033 | 82 | 0.902 | 0.081 | **5.25E-29** | 25 | -0.450 | 0.196 | 0.022 | 24 | -2.320 | 0.338 | **7.14E-12** |
| FG | 46,186 | 82 | 0.083 | 0.017 | **9.28E-07** | 25 | 0.008 | 0.040 | 0.836 | 24 | -0.080 | 0.072 | 0.266 |
| FI | 38,238 | 82 | 0.193 | 0.018 | **3.00E-28** | 25 | 0.119 | 0.041 | 0.00384 | 24 | -0.493 | 0.075 | **4.36E-11** |
| From GIANT: BMI: Body-mass index; WHR: Waist-hip ratio; WC: Waist circumferrence; HIP: hip circumferrence; HT: Height; WT: Weight; From Chu et al. 2017: VAT: Visceral adipose tissue volume; SAT: Subcutaneous adipose tissue volume; PAT: Pericardial adipose tissue volume; From CARDIOGRAM: CAD: Coronary Artery Disease; MI: Myocardial Infarction; From GLGC: HDL-C: High-Density-Lipoprotein-Cholesterol; LDL-C: Low-Density-Lipoprotein-Cholesterol; TG: Triglycerides; From DIAGRAM: T2D: Type 2 Diabetes; From MAGIC: FG: Fasting Glucose; FI: Fasting Insulin | | | | | | | | | | | | | |

## Supplementary Table 2. Characterization of the four adiposity genetics classes with regard to anthropometry, fat depots, metabolic consequences, and implicated pathways. The table summarizes the findings from association enrichment analyses (Table 1, statistical significance at P_Binomial_<0.05/168) and genetic risk score (GRS) analysis (Supplementary Table 1, statistical significance P_GRS_<0.05/60) based on genetic association data for measures of anthropometry, fat depots, and metabolic parameters (using consortium data of GIANT, Ectopic fat trait, DIACORE; MAGIC, GLGC, CARDIoGRAMplusC4D, and UKBiobank) and gene expression analyses using DEPICT and FUMA (Fig. 6, Supplementary Data 9-13, statistical significance at FDR<5% for DEPICT, at Bonferroni-corrected α-level for FUMA). Significant association enrichment analyses results supported by GRS analysis results were denoted as +, -, or 0. Inconclusive results (non-significant, but borderline enrichment in both directions) are noted. One finding was inconsistent between enrichment and GRS analysis (PAT in *BMI+WHR+*). All directions are aligned to the BMI-increasing allele, where appropriate, and for the WHR-decreasing allele for *WHRonly-* (hip-increasing allele for all).

|  | ***BMI+WHR+*** | ***BMIonly+*** | ***WHRonly-*** | ***BMI+WHR-*** |
| --- | --- | --- | --- | --- |
| Anthropometry | all + (waist, hip, weight) | all + (waist, hip, weight) | waist -, hip +, weight 0 | waist 0, hip+, weight + |
| Impedance fat measures | all + (body/trunk/leg fat) | all + (body/trunk/leg fat) | body/trunk/leg fat inconclusive | all + (body/trunk/leg fat) |
| Imaging fat measures | SAT +, VAT+ PAT and VAT/SAT inconclusive | SAT +, VAT 0, PAT 0;  VAT/SAT 0 | VAT/SAT - , SAT 0, VAT 0 | SAT + , VAT 0 , PAT - ;  VAT/SAT - |
| Lipids, FI | HDL-C -, TG +, FI + | inconclusive | lipids -, FI inconclusive | lipids -, FI - |
| T2D, CAD | T2D +, CAD +  **T2D OR=2.46, CAD OR=1.53** | T2D 0, CAD inconclusive | T2D, CAD inconclusive (tendency to -) | T2D -, CAD -  **T2D OR=0.10, CAD OR=0.43** |
| Gene expression enrichment | CNS | CNS | digestive systems, uterus, adipose tissue | adipose tissue |
| Example loci | *FTO*, *MC4R* | *TCF7L2*, *FTO*, *MC4R* | *RSPO3*, *PPARG* | *GRB14*-*COBLL1* |
| General description of adiposity subtype | heavy with strong belly, VAT and SAT increase | heavy overall, no VAT increase, SAT-increase on hip AND waist: hip+waist+ | Fat redistributor: hip+ waist-, (some increased bone growth) | strong hip; SAT-increase on hip only, waist 0  (some PAT-avoider) |
| Cardio-metabolic implication | metabolically unfavourable adiposity | metabolically neutral (except *TCF7L2*) | Inconclusive (tendency towards favourable) | Metabolically rather favourable adiposity |
| Speculated mechanisms | adiposity from positive energy balance (CNS) with predis-position for metabolically unfavourable VAT storage | adiposity from positive energy balance (CNS) with predis-position for metabolically neutral SAT storage (except *TCF7L2*) | VAT/SAT redistribution, no enhanced fat mass (opposite allele: lipodystrophy-like) | adipose-tissue triggered SAT-increase on hip with favourable effect on metabolic health |

# REFERENCES

1. Locke, A.E. *et al.* Genetic studies of body mass index yield new insights for obesity biology. *Nature* **518**, 197-206 (2015).

2. Shungin, D. *et al.* New genetic loci link adipose and insulin biology to body fat distribution. *Nature* **518**, 187-96 (2015).

3. Canela-Xandri, O., Rawlik, K. & Tenesa, A. An atlas of genetic associations in UK Biobank. *bioRxiv* (2017).

4. Lotta, L.A. *et al.* Corrigendum: Integrative genomic analysis implicates limited peripheral adipose storage capacity in the pathogenesis of human insulin resistance. *Nat Genet* **49**, 317 (2017).

5. Lotta, L.A. *et al.* Integrative genomic analysis implicates limited peripheral adipose storage capacity in the pathogenesis of human insulin resistance. *Nat Genet* **49**, 17-26 (2017).

6. Yaghootkar, H. *et al.* Genetic Evidence for a Link Between Favorable Adiposity and Lower Risk of Type 2 Diabetes, Hypertension, and Heart Disease. *Diabetes* **65**, 2448-60 (2016).
