# Supplementary material for: A joint view on genetic variants for adiposity differentiates subtypes with distinct metabolic implications
Source: Nat Commun. 2018 May 16;9:1946. doi: 10.1038/s41467-018-04124-9 (PMC5956079; doi:10.1038/s41467-018-04124-9)
Supplement: Supplementary file 2 — Description of Additional Supplementary Files [file 41467_2018_4124_MOESM2_ESM.pdf]

Description of Additional Supplementary Files

Supplementary Data 1. Obesity traits association results for the 159 identified lead variants. For the 159 identified lead variants, the table shows association results for BMI, WHR and WHRadjBMI. The data shown is based on sex-combined, European-ancestry meta-analyses from the GIANT consortium (see Online Methods for details). Column Region.Signal ID shows a unique identifier for the independent signals (+/- 500kb and r2<0.1) with first number of the ID referring to the larger independent region (+/-500kb) and the second number referring to the independent signal (r2<0.1) within a larger region. Columns W-'AJ' show results from a reclassification of variants based on varying classification thresholds.

Supplementary Data 2. WHR and BMI associations in UK Biobank for the 53 WHRadjBMI-derived variants from GIANT meta-analyses. Shown are the BMI and the WHR effects in UK Biobank (N = 336,107) for the 53 genome-wide significant WHRadjBMI-scan derived variants from the GIANT meta-analysis. The results were downloaded from the GeneAtlas (Canela-Xandri et al. An atlas of genetic associations in UK Biobank. bioRxiv 176834; http://geneatlas.roslin.ed.ac.uk/downloads/?traits=92) for WHR and from the Neale Lab website (https://sites.google.com/broadinstitute.org/ukbbgwasresults/) for BMI. We used a proxy rs9630424 for rs8042543 (r2=0.92) for the lookup of WHR association results because rs8042543 was not available in the downloaded WHR data. The six variants missing an alternative and more stringent classification threshold (PWHR>0.05/159) in the sensitivity classification analyses (**Supplementary Data 1**) are marked in red.

Supplementary Data 3. Sex-specific obesity traits association results for the 159 identified lead variants. For the 159 identified lead variants, the table shows sex-specific association results for BMI, WHR and WHRadjBMI. The data shown is based on sex-specific, European-ancestry meta-analyses from the GIANT consortium (see Online Methods for details).

Supplementary Data 4. Anthropometric traits association results for the 159 identified lead variants. For the 159 identified lead variants, the table shows association results for waist circumference (WC), hip circumference (HIP), weight (WT) and height (HT). The data shown is based on sex-combined, European-ancestry meta-analyses from the GIANT consortium (see Methods for details).

Supplementary Data 5. Impedance measure association results for the 159 identified lead variants. For the 159 identified lead variants, the table shows association results for impedance-based measures of total, leg and trunk fat. The data shown is based on sex-combined association analyses using UK Biobank data (see Online Methods for details).

Supplementary Data 6. Ectopic fat traits association results for the 159 identified lead variants. For the 159 identified lead variants, the table shows association results for computed tomography (CT) and magnetic resonance imaging (MRI) derived measures of visceral adipose tissue (VAT), subcutaneous adipose tissue (SAT), pericardial adipose tissue (PAT) and for the ratio of VAT/SAT. The data shown is based on publically available data from the Ectopic fat traits consortium (see Methods for details).

Supplementary Data 7. Cardio-metabolic traits and diseases association results for the 159 identified lead variants. For the 159 identified lead variants, the table shows association results for coronary artery disease (CAD), myocardial infarction (MI), high-density lipoprotein cholesterol (HDL-C), low-density lipoprotein cholesterol (LDL); triglycerides (TG); fasting glucose (FG), fasting insulin

(FI) and type 2 diabetes (T2D). The data shown is based on publically available data from the DIAGRAM, CARDIOGRAMPLUSC4D, MAGIC and GLGC consortia (see Methods for details).

Supplementary Data 8. Scan-specific DEPICT analyses results. The table shows results from DEPICT tissues, cell type and physiological system enrichment analysis that were conducted separately for variants detected by the BMI-scan, the WHR-scan or the WHRadjBMI-scan (P < 10-5 in the respective GWAS-only meta-analysis results from GIANT). Tissues, cell types and systems that are significantly enriched (FDR<5%) for expression of genes harbouring the identified loci are marked in bold and green.

Supplementary Data 9. Class-specific DEPICT analyses results. The table shows results from DEPICT tissues, cell type and physiological system enrichment analysis that were conducted separately for variants pertaining to the four classes BMI+WHR+, BMIonly+, WHRonly- and BMI+WHR- (using the same variants as in **Supplementary Data 8**, but applying our unsupervised classification). Tissues, cell types and systems that are significantly enriched (FDR<0.05) for expression of genes harbouring the associated loci of a specific class are marked in bold and green.

Supplementary Data 10. Sensitivity analysis for DEPICT focussing on WHRonly- variants after exclusion of height associated variants. The table shows results from DEPICT analysis that were conducted based on WHRonly- variants after exclusion of 697 genetic regions known to be robustly associated with human height14 (removing all WHR-only variants within <250kB around the 697 height-associated lead variants). Tissues, cell types and systems that are significantly enriched (FDR<0.05) for expression of genes harbouring the associated loci of a specific class are marked in bold and green.

Supplementary Data 11. Sensitivity analysis for DEPICT focussing on WHRonly- variants after exclusion of variants harbouring the RSPO3 gene. The table shows results from DEPICT analysis that were conducted based on WHRonly- variants after exclusion of variants harbouring the RSPO3 gene (+/-1Mb). Tissues, cell types and systems that are significantly enriched (FDR<0.05) for expression of genes harbouring the associated loci of a specific class are marked in bold and green.

Supplementary Data 12. Sensitivity analysis for DEPICT focussing on WHRonly- variants and using a distance-only locus definition criterion. The table shows results from DEPICT analysis that were conducted based on WHRonly- variants that were clumped for DEPICT based on a distance-only criterion (+-500kb) without additional reduction based on r2. Tissues, cell types and systems that are significantly enriched (FDR<0.05) for expression of genes harbouring the associated loci of a specific class are marked in bold and green.

Supplementary Data 13. Class-specific FUMA tissue enrichment results. The table shows results from class-specific FUMA tissue specificity analyses of associated variants (P<10-5 for BMI, WHR or WHRadjBMI within GWAS-only meta-analyses of GIANT, excluding Metabochip data). FUMA pre-calculated sets of differentially expressed genes (DEG) for 53 different tissues from the GTEx consortium v6 data. A two-sided t test was applied to each DEG set per tissue versus all remaining and results were Bonferroni corrected based on the number of tests. Genes with a Bonferroni-corrected p-value ≤ 0.05 and absolute log fold change ≥ 0.58 are highlighted in green and bold.

Supplementary Data 14. Overlap of the here identified favorable adiposity variants with previous work from Lotta et al 2017 and Yaghootkar et al 2016. The table shows all variants from the 159 Winkler et al, 53 Lotta et al and 11 Yaghootkar et al variants that showed a favorable disease profile (= nominal significant BMI-increasing AND favorable effects on any of the disease; excluding variants with opposite nominal effects on T2D and CAD).
